# Supplementary material for: Complexome profiling of the Chlamydomonas psb28 mutant reveals TEF5 as an early PSII assembly factor
Source: Plant Cell. 2025 Mar 17;37(6):koaf055. doi: 10.1093/plcell/koaf055 (PMC12164590; doi:10.1093/plcell/koaf055)
Supplement: koaf055_Supplementary_Data [file koaf055_supplementary_data.zip › psb28_interactive.zip!/plots/intensity/qualityPlots/peptides/LHCA9_Cre07.g344950.t1.2.html]

### Cre07.g344950.t1.2

Trivial name: LHCA9  
SeqCoverage [median]: 63.40  
Mass: 22.84 kDa  
  
 

| Sequence | MissedCleavage | Mass [Da] | wtIntensity | muIntensity | Score |
| --- | --- | --- | --- | --- | --- |
| EYDIPAQALTPIEFIVMGFLEIKR | 1 | 2792.48 | 4755.26 | 0.00 | 98.21 |
| EYDIPAQALTPIEFIVMGFLEIKRYQGFK | 2 | 3415.79 | 678.23 | 0.00 | 48.34 |
| GALAGDNGFDPLGLGQDEGR | 0 | 1957.91 | 242564.19 | 104573.50 | 323.59 |
| GALAGDNGFDPLGLGQDEGRLK | 1 | 2199.09 | 139913.86 | 109129.63 | 180.63 |
| LKWYAEAEK | 1 | 1136.59 | 71967.74 | 24961.21 | 152.54 |
| NITYYLTHLPETLGSA | 0 | 1791.90 | 647671.75 | 354468.12 | 244.72 |
| PTWLPGLNPPAHLK | 0 | 1539.86 | 120197.73 | 77578.45 | 172.88 |
| QTGTSGFINSFPFDPAGMNSPSMATK | 0 | 2689.22 | 295833.66 | 102713.63 | 155.61 |
| RYQGFK | 1 | 797.42 | 565.26 | 9580.25 | 130.01 |
| TQPIEGLTAHLADPFGK | 0 | 1793.93 | 1263914.53 | 980137.23 | 125.96 |
| TQPIEGLTAHLADPFGKNITYYLTHLPETLGSA | 1 | 3567.82 | 2941.63 | 0.00 | 32.48 |
| WYAEAEK | 0 | 895.41 | 66391.94 | 7107.71 | 107.32 |
| WYAEAEKTNGR | 1 | 1323.62 | 15969.67 | 5021.80 | 89.40 |
|  |  |  | 2873365.4 | 1775271.5 |  |
